# Circulating MicroRNAs: Biogenesis and Clinical Significance in Acute Myocardial Infarction

**DOI:** 10.3389/fphys.2020.01088

**Published:** 2020-09-03

**Authors:** Lei Zhang, Han Ding, Yuan Zhang, Yin Wang, Wenjie Zhu, Peifeng Li

**Affiliations:** ^1^Institute for Translational Medicine, The Affiliated Hospital of Qingdao University, Qingdao University, Qingdao, China; ^2^The Affiliated Hospital of Qingdao University, Qingdao University, Qingdao, China

**Keywords:** acute myocardial infarction, diagnosis, circulating microRNAs, biomarkers, challenges

## Abstract

Acute myocardial infarction (AMI) causes many deaths around the world. Early diagnosis can prevent the development of AMI and provide theoretical support for the subsequent treatment. miRNAs participate in the AMI pathological processes. We aim to determine the early diagnostic and the prognostic roles of circulating miRNAs in AMI in the existing studies and summarize all the data to provide a greater understanding of their utility for clinical application. We reviewed current knowledge focused on the AMI development and circulating miRNA formation. Meanwhile, we collected and analyzed the potential roles of circulating miRNAs in AMI diagnosis, prognosis and therapeutic strategies. Additionally, we elaborated on the challenges and clinical perspectives of the application of circulating miRNAs in AMI diagnosis. Circulating miRNAs are stable in the circulation and have earlier increases of circulating levels than diagnostic golden criteria. In addition, they are tissue and disease-specific. All these characteristics indicate that circulating miRNAs are promising biomarkers for the early diagnosis of AMI. Although there are several limitations to be resolved before clinical use, the application of circulating miRNAs shows great potential in the early diagnosis and the prognosis of AMI.

## Introduction

Cardiovascular disease is a major cause of human morbidity and mortality. Acute myocardial infarction (AMI) is one of the most serious cardiovascular diseases that occurs worldwide. Early and prompt diagnosis is of great importance to control AMI development and carry out proper treatments to reduce mortality. Currently, some biomarkers from circulatory systems, mainly the blood circulatory system, are widely used in the clinical diagnosis of AMI, such as troponins (TnI/TnT), CK-MB and myoglobin (Ambros, 2004). These biomarkers are non-invasive, making continuous and real-time sampling feasible. Therefore, the disease condition can be constantly monitored. Circulating biomarkers of myocardial damage, especially cardiac-specific troponin, show different concentrations in AMI patients and healthy people. However, troponins are not specific biomarkers for AMI; they can also be detected in severe heart failure, hypothyroidism, septic shock, chronic kidney failure and so on (Jensen et al., 2007; Rosjo et al., 2011). Additionally, the increase of troponins is delayed by at least 3.5 h after AMI onset (Van de Werf et al., 2008), implying that troponins are not effective for the early diagnosis of AMI. Troponins, CK-MB and myoglobin are all proteins that may have limited diagnostic value owing to the confounding effects of the patient’s genetic background, age, lifestyle, medication and so on (Chen et al., 2008). Consequently, there is a clinical need for earlier biomarkers with high specificity and sensitivity.

microRNAs (miRNAs) have various biological and pathological functions with tissue or cell specificity. miRNAs have also been discovered in circulatory systems, opening up new possibilities and opportunities for circulating miRNAs to be potential biomarkers for the diagnosis of cardiovascular diseases (Wang R. et al., 2011; Zhang L. et al., 2018). In this review, we will summarize the available knowledge and discoveries related to circulating miRNAs in AMI diagnosis, with a particular focus on their advantages, and explore the potential clinical use of circulating miRNAs as early and promising diagnostic biomarkers for AMI.

## MiRNAs Relevant to AMI and Diagnostic Strategies for AMI

Acute myocardial infarction is usually caused by the blockage or decrease in the blood flow from moving in the heart. The most typical symptom of AMI is chest pain or discomfort which can radiate to the arm, shoulder, neck, back, jaw or abdomen. AMI consists of several types including unstable angina (UA), STEMI and NSTEMI (Boateng and Sanborn, 2013). The occurrence of AMI brings damage to the heart muscles including cardiac injury and ischemic and hypoxic stress. Most MIs occur due to the blockage by the rupture of atherosclerotic plaques in the coronary arteries (Boateng and Sanborn, 2013). A small number of MIs occur due to coronary artery spasms (Boateng and Sanborn, 2013).

**FIGURE 1:**
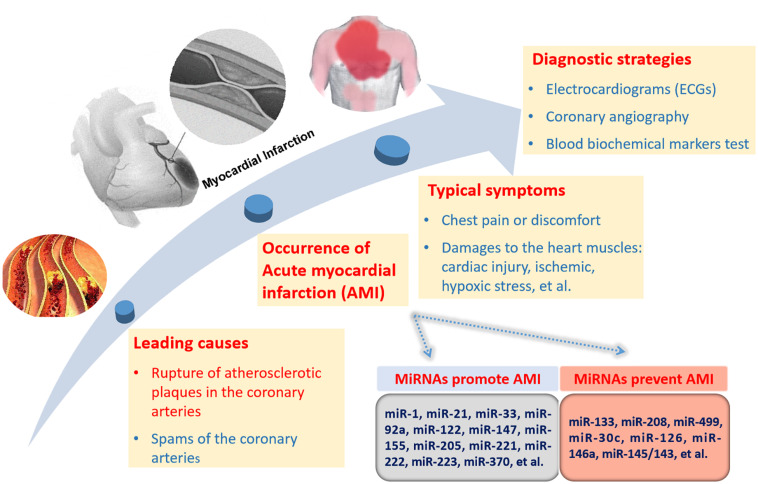
miRNAs related to AMI progression and diagnostic strategies for AMI. There are two causes of AMI with the rupture of atherosclerotic plaques in the coronary arteries as the leading cause. The miRNAs promoting or preventing the formation of AMI are briefly listed. Direct pathological reactions mainly consist of chest pain and chest discomfort. The internal pathological damages are comprised of cardiac injuries, ischemic, hypoxic stress, and so on. There are three diagnostic strategies for AMI, including electrocardiograms (ECGs), coronary angiography and biochemical markers test in blood.

The formation and rupture of atherosclerotic plaques are late symptoms of atherosclerosis. A variety of miRNAs contribute to atherosclerosis. The progression of AMI and the related miRNAs are selectively and simply described in Figure 1. miR-21 and miR-92a promote endothelial damage and dysfunction (Bonauer et al., 2009; Loyer et al., 2014). miR-223, miR-155, miR-205, and miR-147 take part in endothelial cell inflammation (Liu et al., 2009; Son et al., 2013; Du et al., 2014; Tabet et al., 2014; Wei Y. et al., 2015). miR-122 and miR-370 facilitate the formation of total cholesterol and triglycerides (Esau et al., 2006; Iliopoulos et al., 2010). miR-221 and miR-222 stimulate vascular smooth muscle cell (VSMC) proliferation and migration (Liu et al., 2012). miR-33 increases plaque size and lipid content (Rayner et al., 2010). All the above miRNAs can facilitate the formation of atherosclerosis. miR-126 promotes endothelial cell proliferation (Kuhnert et al., 2008). miR-30c can downregulate lipid synthesis and lipoprotein secretion (Duisters et al., 2009). miR-146a functions by inhibiting the inflammatory response and the lipid accumulation induced by the oxidized low-density lipoprotein (Yang et al., 2011). miR-145/143 can increase the plaque stability and reduce the plaque size and necrotic core area. These miRNAs will prevent atherosclerosis development (Cordes et al., 2009). In addition to the above miRNAs, there are many other miRNAs involved in this process. Overall, miRNAs take part in every event of atherosclerosis development, indicating the important role of miRNAs in AMI.

Currently, diagnostic strategies for AMI include ECGs (electrocardiograms), coronary angiography and biochemical blood marker tests. ECGs can record the beating and rhythm of the heart, but they cannot reveal the pathological changes inside the heart. Coronary angiography is a safe and reliable diagnostic technology. However, it is invasive and conditional. Compared with ECGs and coronary angiography, biochemical markers are non-invasive, innocuous and harmless. Due to the intimate association of miRNAs with the formation and rupture of atherosclerotic plaques, the diagnostic role of circulating miRNAs in AMI is greatly expected.

## The Biogenesis of Circulating MiRNAs

**FIGURE 2:**
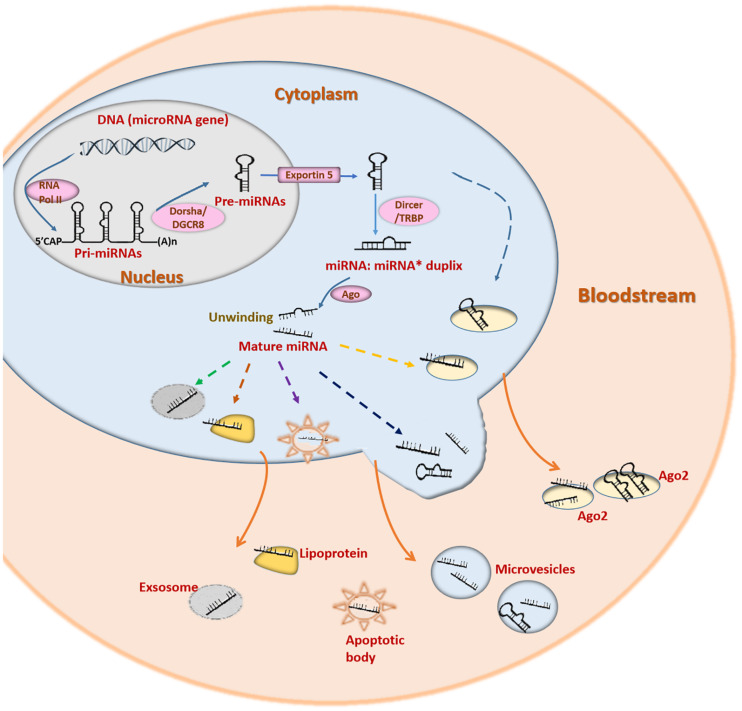
The underlying mechanisms of miRNA biogenesis, cellular release and circulation. Pri-miRNAs are generated from miRNA genes with the help of RNA polymerase II and cleaved by Drosha/DGCR8 to form pre-miRNAs. Pre-miRNAs are then transported into cytoplasm and cleaved by Dicer/TRBP. The miRNA duplexs produced are then translated into mature miRNAs. However, only one strand of the miRNA duplex is translated into the mature miRNA. Both pre-miRNAs and mature miRNAs can be released into the bloodstream by several pathways. miRNAs can be released by association with particles, such as lipoprotein complexes (HDL) and RNA-binding proteins (Ago2), or by being packaged in vesicles (exosomes, microvesicles, and apoptotic bodies).

miRNAs are a large class of endogenous small non-coding RNAs (18-25 nucleotides). miRNAs target the 3′- untranslated region of mRNAs to inhibit mRNA translation or induce degradation (Ambros, 2004). Most miRNAs are generated from the introns of functional genes (Sibley et al., 2012). miRNA genes are transcribed into primary miRNAs (pri-miRNAs) by RNA polymerase II. Pri-miRNAs are processed and cleaved into precursor miRNAs (pre-miRNAs) by Drosha (RNase III enzyme) and DGCR8 (DiGeorge syndrome critical region 8) (Figure 2) (Han et al., 2004). Pre-miRNAs are subsequently exported into the cytoplasm by exportin-5 (Yi et al., 2003). The cytoplasmic pre-miRNAs are cleaved by Dicer (an RNase III enzyme complex) to generate mature miRNAs (Figure 2) (Madina et al., 2011). A single mature miRNA can target one or more genes, while one protein-coding gene can be targeted by various miRNAs (Park et al., 2011). This enables miRNAs to function in a variety of physiological and pathological processes (Wu et al., 2011; Zhao Y. et al., 2017).

Most miRNAs are located intracellularly, but there are extracellular miRNAs in plasma, serum and other body fluids, which are called circulating miRNAs (Weber et al., 2010). Despite RNases in body fluids, circulating miRNAs show stable existence. Moreover, circulating miRNAs are stable even under extremely harsh conditions, such as repeated boiling, very high or low pH, freezing-thawing cycles and room temperature storage (Turchinovich et al., 2011).

The stability of circulating miRNAs is relevant to the mechanisms by which they are produced and transported (Figure 2). Circulating miRNAs are secreted into circulation mainly in the following two ways: encapsulated in lipid vesicles (exosomes, microvesicles and apoptotic bodies) and being associated with RNA binding proteins (nucleophosmin 1, NPM1 and Argonaute protein 2, Ago2) or lipoprotein complexes (Fritz et al., 2016). The majority of circulating miRNAs are transported with RNA binding proteins. Ago2 is a key effector protein of RISC (Arroyo et al., 2011). NPM1 is located in the nucleus and participates in the nuclear exportation of the ribosome and nucleolin (Maggi et al., 2008). Ago2 and NPM1 can combine with miRNAs to form a highly stable complex, protecting miRNAs from degradation by RNases (Wang K. et al., 2010). Lipoprotein complexes mainly include HDL and low-density lipoprotein (LDL). They can also transport endogenous miRNAs in association with each other (Vickers et al., 2011). Lipid vesicles are responsible for only a small portion of circulating miRNA transportation (Fritz et al., 2016). Lipid vesicles have different sizes, origins and release modes. Exosomes (30–100 nm) originate from endosomes and are secreted into circulation when the multivesicular bodies are fused with the plasma membrane (Raposo and Stoorvogel, 2013). Microvesicles (0.1–1 mm) are released through outward budding (Fritz et al., 2016). Apoptotic bodies (0.5–2 mm) are secreted during apoptosis (Fritz et al., 2016). Lipid vesicles in the circulation have been discovered to function in cell-to-cell communication by fusing with the membrane of target recipient cells (Fritz et al., 2016). The miRNAs included in the lipid vesicles could be taken up by recipient cells even over a long distance, enabling cell-to-cell communication (Barile et al., 2014). miRNAs transported by HDL can also regulate the gene expression in distant cells (Vickers et al., 2011).

## Circulating MiRNAs Function as Diagnostic and Prognostic Biomarkers for AMI

miRNAs participate in a variety of physiological and pathological processes. The abnormal expression of intracellular miRNAs is relevant to various diseases, including cardiovascular diseases, such as AMI, endothelial dysfunction and angiogenesis (Cordes et al., 2009; Weber et al., 2010; Peng et al., 2014; Kondkar and Abu-Amero, 2015). Circulating miRNAs have been reported to exhibit altered expression levels in cancer, cardiovascular diseases and so on (Fichtlscherer et al., 2011; Karakas et al., 2017).

An ideal and mature diagnostic biomarker for AMI should meet many criteria, including non-invasive sampling, high specificity and sensitivity, content elevation in the early stage of certain diseases and fast and accurate detection. Circulating miRNAs have several advantages. The way by which circulating miRNAs are harvested is harmless and simple. They are very stable and can withstand extreme conditions. In addition, they are tissue- and disease-specific. Furthermore, they can be detected quantitatively by real-time PCR, microarray and so on. Therefore, circulating miRNAs have great potential to be AMI diagnostic biomarkers. To date, many studies have revealed the promising role of circulating miRNAs in the early diagnosis of AMI (Tables 1, 2).

**TABLE 1:** Heart- and muscle- enriched miRNAs as biomarkers of AMI.

miRNA	Normalization	Control/Case	Regulation	Extraction method	Clinical value	References
**miR-1**	U6 snRNA	66/93 MI	Up	mirVana PARIS isolation kit (Ambion)	Diagnosis (AUC: 0.7740)	Ai et al., 2010, #31
	N/A	20/31 AMI	Up	miRNAs Isolation Kit (ShortHills)	Diagnosis (ROC analysis not performed)	Cheng et al., 2010, #28
	miR-17-5p	17/33 STEMI	Up	mirVana PARIS isolation kit (Ambion)	Diagnosis (ROC analysis not performed)	D’Alessandra et al., 2010, #29
	miRNA spike-in	11/25 MI	Up	TRIzol Reagent	Diagnosis (AUC: 0.98)	Gidlof et al., 2011, #32
	N/A	42/29 ACS	Up	TRIzol Reagent	Diagnosis (AUC: 0.777)	Kuwabara et al., 2011, #30
	miR-16	20/19 UA, 34 SA	Up	TRIzol Reagent	Diagnosis (AUC for SA: 0.918; UA:0.920)	D’Alessandra et al., 2013, #45
	Cel-miR-39	32/67 AMI	Up	TRIzol Reagent	Diagnosis (AUC: 0.8265)	Li et al., 2013, #36
	miR-17	99/92 NSTEMI	Up	Not mentioned	Diagnosis (ROC analysis not performed)	Olivieri et al., 2013, #35
	Cel-miR-39	72/70 AMI	Up	TRIzol Reagent	Diagnosis (AUC: 0.81)	Liu X. et al., 2015, #34
**miR-133a/b**	miR-17-5p	17/33 STEMI	Up	mirVana PARIS isolation kit (Ambion)	Diagnosis (ROC analysis not performed)	D’Alessandra et al., 2010, #29
	N/A	42/29 ACS	Up	TRIzol Reagent	Diagnosis (AUC: 0.932)	Kuwabara et al., 2011, #30
	miRNA spike-in	11/25 MI	Up	TRIzol Reagent	Diagnosis (AUC: 0.86)	Gidlof et al., 2011, #32
	Cel-miR-54	0/117 UA, 131 NSTEMI, 196 STEMI	Up	Master Pure RNA Purification Kit	Diagnosis or prognosis (AUC: 0.57)	Widera et al., 2011, #33
	U6 snRNA	28/51 AMI	Up	TRIzol Reagent	Diagnosis (AUC for plasma and whole blood: 0.890 and 0.810)	Wang R. et al., 2011, #27
	miR-16	20/19 UA, 34 SA	Up	TRIzol Reagent	Diagnosis of 133a (AUC for UA:0.906;SA:0.772). Diagnosis of 133b (AUC for UA:0.776;SA:0.844)	D’Alessandra et al., 2013, #45
	Cel-miR-39	32/67 AMI	Up	TRIzol Reagent	Diagnosis (AUC: 0.9468)	Li et al., 2013, #36
	U6 snRNA	127/13 AMI, 176 AP	Up	TRIzol Reagent	Diagnosis (ROC analysis not performed)	Wang et al., 2013, #119
	miR-17	99/92 NSTEMI, 81 CHF	Up	Not mentioned	Diagnosis (ROC analysis not performed)	Olivieri et al., 2013, #35
	miR-16	110/76 AMI	Up	miRNeasy Mini Kit (Qiagen)	Diagnosis (AUC: 0.912)	Peng et al., 2014, #37
**miR-208a/b**	Cel-miR-39	30/33 AMI	Up	TRIzol Reagent	Diagnosis (AUC: 0.965)	Wang G. K. et al., 2010, #40
	Cel-miRs (3×)	36/32 AMI	Up	mirVana PARIS isolation kit (Ambion)	Diagnosis (ROC analysis not performed)	Corsten et al., 2010, #44
	miRNA spike-in	11/25 MI	Up	TRIzol Reagent	Diagnosis (AUC: 1.0)	Gidlof et al., 2011, #32
	Cel-miR-54	0/117 UA,131 NSTEMI, 196 STEMI	Up	Master Pure RNA Purification Kit	Diagnosis and prognosis (AUC:0.57)	Widera et al., 2011, #33
	Cel-miR-39	32/67 AMI	Up	TRIzol Reagent	Diagnosis (AUC: 0.8899)	Li et al., 2013, #36
	SV40 spike-in	243/116 AMI	Up	TRIzol Reagent	Diagnosis and prognosis (AUC: 0.780)	Lv et al., 2014, #42
	Cel-miR-39	72/70 AMI	Up	TRIzol Reagent	Diagnosis (AUC: 0.72)	Liu X. et al., 2015, #34
**miR-499**	Spike-in RNA	10/14 ACS, 15 CHF	Up	mirVana PARIS Kit (Ambion)	Diagnosis (ROC analysis not performed)	Adachi et al., 2010, #48
	miR-17-5p	17/33 STEMI	Up	mirVana PARIS isolation kit (Ambion)	Diagnosis (ROC analysis not performed)	D’Alessandra et al., 2010, #29
	Cel-miRs (3×)	36/32 AMI	Up	mirVana PARIS isolation kit (Ambion)	Diagnosis (ROC analysis not performed)	Corsten et al., 2010, #44
	miRNA spike-in	11/25 MI	Up	TRIzol Reagent	Diagnosis (AUC: 0.99)	Gidlof et al., 2011, #32
	Cel-miR-39	32/67 AMI	Up	TRIzol Reagent	Diagnosis (AUC: 0.8841)	Li et al., 2013, #36
	miR-17	99/92 NSTEMI, 81 CHF	Up	not mentioned	Diagnosis (AUC: 0.86)	Olivieri et al., 2013, #35
	Median Ct	89/28 AMI	Up	mirVana PARIS isolation kit (Ambion)	Diagnosis and prognosis (AUC: 0.939)	Yao et al., 2014, #49
	Mean *C*t	30/73 ACS	Up	mirVana PARIS isolation kit (Ambion)	Diagnosis (ROC analysis not performed)	Chen et al., 2015, #47
	Cel-miR-39	72/70 AMI	Up	TRIzol Reagent	Diagnosis (AUC: 0.88)	Liu X. et al., 2015, #34
	U6 snRNA	100/142 AMI	Up	mirVana PARIS isolation kit (Ambion)	Diagnosis (AUC: 0.86)	Zhang L. et al., 2015, #46
	U6 snRNA	25/37 UA, 48 NSTEMI	Up	RNA extraction kit (Qiagen)	Diagnosis (AUC for UA: 0.98; NSTEMI: 0.97)	Shalaby et al., 2016, #50

*MI, myocardial infarction; AMI, acute myocardial infarction; NSTEMI, non-ST-segment-elevation myocardial infarction; STEMI, ST-segment-elevation myocardial infarction; ACS, acute coronary syndrome; UA, unstable angina pectoris; SA, stable angina pectoris; AP, angina pectoris; CHF, congestive heart failure; CAD, coronary artery disease; snRNA, small nuclear RNA; Ct, cycle threshold.*

**TABLE 2:** Non-cardiac miRNAs as biomarkers of AMI.

miRNA	Normalization	Control/Case	Regulation	Extraction method	Clinical value	References
**miR-16**	Cel-miRs (3×)	0/150 AMI	Up	mirVana PARIS isolation kit (Ambion)	Diagnosis (ROC analysis not performed)	Devaux et al., 2013a, #57
**miR-19b**	Cel-miR-39	140/140 AMI	Up	miRNeasy Plasma Kit (Qiagen)	Diagnosis (AUC: 0.74)	Li L. et al., 2019, #59
	Cel-miR-39	18/20 AMI	Up	TRIzol Reagent	Diagnosis (AUC: 0.821)	Wang K. J. et al., 2016, #60
**miR-21**	miR-17	99/92 NSTEMI, 81 CHF	Up	Not mentioned	Diagnosis (ROC analysis not performed)	Olivieri et al., 2013, #35
	U6 snRNA	25/38 AMI	Up	total-RNA extraction kit	Diagnosis (ROC analysis not performed)	Wang et al., 2017, #54
	Cel-miR-39	10/66 AMI	Up	RNAprep pure Blood Kit (Tiangen)	Diagnosis (AUC: 0.892	Zhang Y. et al., 2016, #55
**miR-22-5p**	Cel-miR-39	70/66 AMI	Up	TRIzol Reagent	Diagnosis (AUC: 0.975)	Wang et al., 2019, #63
**miR-27a**	Cel-miRs (3×)	0/150 AMI	Up	mirVana PARIS isolation kit (Ambion)	Diagnosis (ROC analysis not performed)	Devaux et al., 2013a, #57
**MiR-30c**	RNU6B-2	20/20 STEMI	Up	miRNeasy Mini Kit (Qiagen)	Diagnosis and prognosis (ROC analysis not performed)	Meder et al., 2011, #66
**miR-30d-5p**	Cel-miR-39	79/230 ACS	Up	TRIzol Reagent	Diagnosis and prognosis (AUC: 0.915)	Jia et al., 2016, #64
**miR-34a**	SV40 spike-in	243/116 AMI	Up	TRIzol Reagent	Diagnosis and prognosis (AUC: 0.738)	Lv et al., 2014, #42
**miR-122**	miR-16	20/19 UA, 34 SA	Up	TRIzol Reagent	Diagnosis (AUC for UA: 0.689; SA: 0.734)	D’Alessandra et al., 2013, #45
	U6 snRNA	39/50 AMI	Up	miRcute miRNA Isolation Kit (Tiangen)	Diagnosis (AUC: 0.855)	Yao et al., 2015, #62
	Cel-miR-39	70/66 AMI	Up	TRIzol Reagent	Diagnosis (AUC: 0.626)	Wang et al., 2019, #63
**miR-124**	GAPDH	45/90 AMI	Up	TRIzol Reagent	Diagnosis (AUC: 0.86)	Guo et al., 2017, #67
**miR-125b-5p**	Cel-miR-39	79/230 ACS	Up	TRIzol Reagent	Diagnosis and prognosis (AUC: 0.879)	Jia et al., 2016, #64
**miR-126**	Average *C*t	820/population-based study	Up	miRNeasy kit (Qiagen)	Diagnosis (ROC analysis not performed)	Zampetaki et al., 2012, #51
	miR-16	20/19 UA, 34 SA	Up	TRIzol Reagent	Diagnosis (AUC for SA: 0.929; UA: 0.867)	D’Alessandra et al., 2013, #45
**miR-134**	SV40 spike-in	30/359 AMI	Up	TRIzol Reagent	Diagnosis and prognosis (AUC: 0.818)	He et al., 2014, #52
	U6 snRNA	20/18 AMI	Up	TRIzol Reagent	Diagnosis (AUC: 0.827)	Wang K. J. et al., 2016, #60
**miR-144-3p**	5 spike-in RNAs	100/112 AMI	Up	miRCURY RNA isolation kit for biofluids (Exiqon)	Diagnosis (Combined AUC: 0.91)	Bye et al., 2016, #69
**miR-145**	RNU6B-2	20/20 STEMI	Up	miRNeasy Mini Kit (Qiagen)	Prognosis (ROC analysis not performed)	Meder et al., 2011, #66
	miR-16	20/19 UA, 34 SA	Up	TRIzol Reagent	Diagnosis (AUC for UA: 0.779; SA: 0.725)	D’Alessandra et al., 2013, #45
**miR-150**	U6 snRNA	110/65 STEMI, 45 NSTEMI	Up	miRNeasy Serum/Plasm Kit (Qiagen)	Diagnosis (AUC for STEMI: 0.639; NSTEMI: 0.734)	Zhang R. et al., 2015, #53
**miR-186-5p**	Cel-miR-39	18/20 AMI	Up	TRIzol Reagent	Diagnosis (AUC:0.796)	Wang K. J. et al., 2016, #60
**miR-199a**	miR-16	20/19 UA, 34 SA	Up	TRIzol Reagent	Diagnosis (AUC for UA: 0.739; SA: 0.821)	D’Alessandra et al., 2013, #45
**miR-210**	U6 snRNA	25/37 UA, 48 NSTEMI	Up	RNA extraction kit (Qiagen)	Diagnosis (AUC for UA: 0.98; NSTEMI: 0.97)	Shalaby et al., 2016, #50
**miR-221-3p**	Two spike-in controls, six housekeeping genes	16/27 AMI	Up	miRNeasy Serum/Plasm Kit (Qiagen)	Diagnosis (AUC: 0.892)	Coskunpinar et al., 2016, #68
**miR-223**	Cel-miR-39	140/140 AMI	Up	miRNeasy Plasma Kit (Qiagen)	Diagnosis (AUC: 0.65)	Li L. et al., 2019, #59
**miR-328**	SV40 spike-in	30/359 AMI	Up	TRIzol Reagent	Diagnosis and prognosis (AUC: 0.887)	He et al., 2014, #52
	U6 snRNA	28/51 AMI	Up	TRIzol Reagent	Diagnosis (AUC for plasma and whole blood samples: 0.702 and 0.872)	Wang R. et al., 2011, #27
**miR-337-5p**	miR-16	20/19 UA, 34 SA	Up	TRIzol Reagent	Diagnosis (AUC for UA: 0.693; SA: 0.796)	D’Alessandra et al., 2013, #45
**miR-423-5p**	miR-17	99/92 NSTEMI, 81 CHF	Up	Not mentioned	Diagnosis (ROC analysis not performed)	Olivieri et al., 2013, #35
**miR-424-5p**	5 spike-in RNAs	100/112 AMI	Up	miRCURY RNA isolation kit for biofluids (Exiqon)	Diagnosis (Combined AUC: 0.91)	Bye et al., 2016, #69
**miR-483-5P**	Cel-miR-39	140/140 AMI	Up	miRNeasy Plasma Kit (Qiagen)	Diagnosis (AUC: 0.70)	Li L. et al., 2019, #59
**miR-485-3p**	miR-16	20/19 UA, 34 SA	Up	TRIzol Reagent	Diagnosis (AUC for SA: 0.851; UA: 0.808)	D’Alessandra et al., 2013, #45
**miR-486**	U6 snRNA	110/65 STEMI, 45 NSTEMI	Up	miRNeasy Serum/Plasm Kit (Qiagen)	Diagnosis (AUC for STEMI: 0.695; NSTEMI: 0.782)	Zhang R. et al., 2015, #53
**miR-660-5p**	5 spike-in RNAs	100/112 AMI	Up	miRCURY RNA isolation kit for biofluids (Exiqon)	Diagnosis (Combined AUC:0.91)	Bye et al., 2016, #69
**miR-663b**	miR-16	110/76 AMI	Up	miRNeasy Mini Kit (Qiagen)	Diagnosis (AUC: 0.611)	Peng et al., 2014, #37
**miR-1291**	miR-16	110/76 AMI	Up	miRNeasy Mini Kit (Qiagen)	Diagnosis (AUC: 0.695)	Peng et al., 2014, #37
**miR-101**	Cel-miRs (3×)	150 AMI	Down	mirVana PARIS isolation kit (Ambion)	Diagnosis (ROC analysis not performed)	Devaux et al., 2013a, #57
**miR-106a-5p**	5 spike-in RNAs	100/112 AMI	Down	miRCURY RNA isolation kit for biofluids (Exiqon)	Diagnosis (Combined AUC: 0.91)	Bye et al., 2016, #69
**let-7g-5p**	5 spike-in RNAs	100/112 AMI	Down	miRCURY RNA isolation kit for biofluids (Exiqon)	Diagnosis (Combined AUC: 0.91)	Bye et al., 2016, #69
**miR-122**	miR-17-5p	17/33 STEM	Down	mirVana PARIS isolation kit (Ambion)	Diagnosis (AUC for UA: 0.689; SA: 0.734)	D’Alessandra et al., 2013, #45
**miR-150**	Cel-miRs (3×)	150 AMI	Down	miRVana PARIS isolation kit (Ambion)	Diagnosis (ROC analysis not performed)	Devaux et al., 2013a, #57
**miR-197**	Average *C*t	773/Population -based study	Down	miRNeasy kit (Qiagen)	Diagnosis (ROC analysis not performed)	Zampetaki et al., 2012, #51
**miR-223**	Average *C*t	773/47 AMI	Down	miRNeasy kit (Qiagen)	Diagnosis (ROC analysis not performed)	Zampetaki et al., 2012, #51
**miR-663b**	RNU6B-2	20/20 STEMI	Down	miRNeasy Mini Kit (Qiagen)	Diagnosis (AUC: 0.94)	Meder et al., 2011, #66
**miR-1291**	RNU6B-2	20/20 STEMI	Down	miRNeasy Mini Kit (Qiagen)	Diagnosis (AUC: 0.91)	Meder et al., 2011, #66

*MI, myocardial infarction; AMI, acute myocardial infarction; NSTEMI, non-ST-segment–elevation myocardial infarction; STEMI, ST-segment–elevation myocardial infarction; ACS, acute coronary syndrome; UA, unstable angina pectoris; SA, stable angina pectoris; AP, angina pectoris; CHF, congestive heart failure; CAD, coronary artery disease; snRNA, small nuclear RNA; Ct, cycle threshold.*

### Heart- and Muscle- Enriched miRNAs

There are more than 200 miRNAs in the heart (Wang et al., 2008; Bauersachs and Thum, 2011), among which miR-1, miR-133a, miR-208a, and miR-499 are the most studied. miR-208a is heart-specific (van Rooij et al., 2007), whereas the other three miRNAs are highly expressed in both the heart and the skeletal muscles (Chen et al., 2006; van Rooij et al., 2009). miR-1, miR-133a, miR-208a and miR-499 were found to be released into the circulation in exosomes from infarcted mouse hearts, and their circulating levels were all increased (Kuwabara et al., 2011). The circulating levels of miR-1, miR-133a, miR-208b, and miR-499-5p were rapidly and significantly increased in rats and adult pigs with AMI (Gidlof et al., 2011; Wang G. K. et al., 2010). In the bloodstream of AMI patients, miR-1, miR-133, miR-208, and miR-499 were all elevated (Table 1). These findings suggest their roles in the diagnosis and prognosis of AMI.

miR-1 was found to deteriorate arrhythmogenesis in infarcted rat hearts by suppressing the generation of functional proteins such as GJA1 and KCNJ2 (Yang et al., 2007). miR-1 was also reported to exacerbate cardiac ischemia-reperfusion injury (Pan et al., 2012). Therefore, miR-1 might aggravate AMI. Cheng et al. (2010) and Wang G. K. et al. (2010) found a rapid increase in circulating miR-1 in rats after the ligation of coronary artery. Circulating miR-1 peaked (over 200-fold increase) at 6 h of AMI onset and returned to the basal level in 3 days, which showed a faster and earlier time course than known biomarkers, such as troponins (Cheng et al., 2010). D’Alessandra et al. (2010) revealed a rise in circulating miR-1 in mice at the 6-h time point and a peak at the 18-h time point after coronary occlusion. Circulating miR-1 was also remarkably increased in AMI patients (Ai et al., 2010; Cheng et al., 2010; D’Alessandra et al., 2010; Wang G. K. et al., 2010). Cheng et al. (2010) detected an ∼100-fold increase in serum miR-1 6 h after AMI. In addition, circulating miR-1 was positively correlated with CK-MB and myocardial infarct size (Cheng et al., 2010). Ai et al. (2010) also detected a significantly higher level of plasma miR-1 in AMI patients. The increased level of miR-1 was negatively associated with arrhythmia but had no correlation with CK-MB/cTnI (Ai et al., 2010). All findings suggest that miR-1 might be a diagnostic biomarker for AMI.

miR-133 has been found to protect cardiomyocytes against myocardial infarction (Yu et al., 2019). Wang G. K. et al. (2010) detected a rapid increase in circulating miR-133a 1 h after AMI in rats. The circulating level of miR-133a peaked at the third hour of symptom onset (∼1,000-fold higher than the baseline). D’Alessandra et al. (2010) established a time course of circulating miRNA levels in AMI mice. The highest levels of circulating miR-133a and miR-133b in mice both appeared 6 h after AMI (∼13-fold increase and ∼5-fold increase, respectively) (D’Alessandra et al., 2010). Increased level of circulating miR-133 were also found in clinical trials. Wang G. K. et al. (2010) revealed a 4.4-fold increase in plasma miR-133a in AMI patients and a positive correlation between plasma miR-133a and cTnT. D’Alessandra et al. (2010) showed a fast elevation of plasma miR-133a and miR-133b which reached the peak in just 517 ± 309 min after the onset of AMI, much earlier than cTnT. Similar findings were also reported by Wang et al. (2013). In a study with a large cohort of ACS patients, Widera et al. (2011) stated that there was an independent association of plasma miR-133a and miR-133b with hsTnT. However, miR-133 could not be a substitute for hsTnT or improve the established diagnostic role of hsTnT. Bauters et al. (2013) illustrated that miR-133a could not be a diagnostic biomarker for the left ventricular remodeling after MI.

miR-208 is present in the heart and thus functions only in the cardiac-specific physiological processes. miR-208 regulates the normal expression of cardiac genes and participates in a series of pathological cardiac processes, such as cardiac remodeling and fibrosis (Kondkar and Abu-Amero, 2015). Decreased level of miR-208 in the heart can lead to ischemia myocardial and reperfusion injury by targeting p21, promoting the formation of AMI (Liu C. et al., 2016). miR-208a is a heart-specific miRNA, so non-cardiac damage and disease have minimal impact on it. This characteristic makes it a superior diagnostic tool for AMI. Wang G. K. et al. (2010) reported significantly increased levels of the plasma miR-1, miR-133a, miR-208a, and miR-499 in AMI rats, in which miR-208a showed the best detection sensitivity and specificity. Plasma miR-208a was undetectable before AMI, but was apparently increased within 1 h after myocardial injury and peaked at the third hour (∼1,000-fold above baseline) (Wang G. K. et al., 2010). In comparison, the elevated levels of blood cTnI were detected 4–8 h after the onset of myocardial injury (Wang G. K. et al., 2010; Bialek et al., 2015). This indicated that miR-208a had greater diagnostic value during the early stage of AMI than did conventional protein markers. Bialek et al. (2015) also confirmed the higher and earlier (3 h after symptom onset) diagnostic value of circulating miRNA-208a than cTnT and CK-MBmass in STEMI patients (6 h after symptom onset). Xiao et al. (2014) illustrated the predictive role of serum miRNA-208a in AMI patients. Liu X. et al. (2015) revealed that miR-208, along with miR-499, displayed a more reliable value than miR-1 in AMI diagnosis. Lv et al. (2014) reported the elevated level of circulating miR-208b in the left ventricular (LV) remodeling after AMI and a positive correlation of miR-208b with the risk of mortality or HF. Widera et al. (2011) confirmed the association of miR-208b with hsTnT in a large cohort of 444 patients with ACS. But they found that the incorporation of miR-208b to hsTnT could not improve the diagnostic value. Some studies reported extremely low concentrations of miR-208a and miR-208b in MI/AMI patients which might make accurate detection difficult and lead to considerable error (Adachi et al., 2010; D’Alessandra et al., 2010).

miR-499 is highly expressed in the heart. miR-499 has been illustrated to attenuate the occurrence of myocardial infarction through inhibiting the activity of calcineurin (Wang J. X. et al., 2011). Wang G. K. et al. (2010) reported an increase of plasma miR-499 in 1 h and the highest level of plasma miR-499 3 h after coronary artery ligation in rats. D’Alessandra et al. (2010) illustrated that miR-499 was extremely sensitive to cardiac damage and the most sensitive among four cardiac miRNAs in mice. The plasma level of miR-499 obviously increased just 15 min after coronary ligation, almost simultaneous to the change in cTnI (D’Alessandra et al., 2010). However, it took it a much longer time for plasma level of miR-499 (∼24 h) to reach a peak than cTnI (∼6 h) (D’Alessandra et al., 2010). The elevated levels of circulating miR-499 in AMI patients have been described by many clinical studies (Adachi et al., 2010; Xiao et al., 2014; Yao et al., 2014; Chen et al., 2015; Liu X. et al., 2015; Zhang L. et al., 2015; Shalaby et al., 2016). Li et al. (2013) illustrated that miR-499 was more accurate than cTnT for the diagnosis of non-STEMI among elderly patients. Olivieri et al. (2013) compared the ability of cTnT and miR-499 to distinguish non-STEMI and congestive HF. Their study indicated that miR-499 had greater discriminatory power than cTnT (Olivieri et al., 2013). Chen et al. (2015) proposed that the elevation of miR-499 was correlated with the severity of AMI. Zhang L. et al. (2015) showed that serum miR-499 was positively associated with both CK-MB and cTnI. Shalaby et al. (2016) found that elevated serum level of miR-499 was associated with UA and STEMI. Yao et al. (2014) suggested the potential role of miR-499 in the mortality risk stratification and in the identification of perioperative AMI in cardiac surgery.

In addition to the cardiac functions, miR-1, miR-133, and miR-499 are also skeletal muscle-enriched. miR-1, miR-133 and miR-499 play pivotal roles in the proliferation and differentiation of skeletal muscles (Chen et al., 2006; Horak et al., 2016; Jiang et al., 2018). The expression levels of miR-1, miR-133, and miR-499-5p were downregulated in the adductor skeletal muscles and ischemic gastrocnemius within 24 h after femoral artery dissection (D’Alessandra et al., 2010). In the children with Duchenne muscular dystrophy, expression levels of miR-1 and miR-133 in serum were significantly increased compared with the healthy controls (Hu et al., 2014). Serum levels of miR-1 and miR-133a were found to be strikingly upregulated in the mouse upon skeletal muscle regeneration induced by muscle injury (Matsuzaka et al., 2014). miR-1 exhibited significantly elevated serum levels in the patients with limb-girdle muscular dystrophy, facioscapulohumeral muscular dystrophy, and becker muscular dystrophy (BMD) compared with controls (Matsuzaka et al., 2014). miR-133a presented markedly upregulated serum expression in BMD patients in contrast to controls (Matsuzaka et al., 2014). However, little is known about the expression of miR-499 in pathological condition of skeletal muscle. As the pathological condition of skeletal muscle influences miR-1 and miR-133, further confirmation of the skeletal muscle of AMI patients should be carried out to exclude skeletal muscle diseases when miR-1 and miR-133 are used as the diagnostic biomarkers. If AMI patients are confirmed to have skeletal muscle disease, a combination of miR-1 and miR-133 with other diagnostic biomarkers can be used, or miR-1 and miR-133 can be substitute for other diagnostic biomarkers.

miR-1, miR-133, and miR-499 also function in other diseases such as cancers, organ injuries and so on. Serum level of miR-1 was significantly increased in the polymyositis (PM)/dermatomyositis (DM) and serum miR-1 might be a promising novel biomarker for predicting PM/DM (Sugiyama et al., 2019). Level of serum miR-1 was elevated in patients with Acute Kawasaki Disease (KD). ROC analysis validated the potential role of serum miR-1 in the diagnosis of KD (Yan et al., 2019). miR-1 also act as tumor suppressor to inhibit the development of a series of cancers (Chang et al., 2015; Xu et al., 2017; Zhang J. et al., 2018). Plasma level of miR-1 was obviously reduced in lung cancer and ROC analysis indicated the diagnostic potential of miR-1 (Sheervalilou et al., 2019). Serum miR-133 level was significantly upregulated and had great potential to be biomarker for diagnosing of Lymphoma associated hemophagocytic syndrome (Li W. et al., 2017). Serum miR-133 was downregulated in patients with breast cancer (Hesari et al., 2018). miR-499 participates in the proliferation and metastasis of different cancers (Li et al., 2016; Zhang X. et al., 2016). miR-499 plays a role in the pathogenesis and progression of traumatic brain injury (TBI). Elevated serum miR-499 has been revealed to be non-invasive biomarkers for the presence and progression of TBI (Yang et al., 2016).

Taken together, the results for the four heart- and muscle-enriched miRNAs demonstrate their value in AMI diagnosis. Circulating levels of the four miRNAs are increased in AMI patients compared to those in the control subjects and display considerable diagnostic value. Compared with CK-MB and troponins, they show earlier alterations in expression after the AMI onset, indicating their higher value in the early diagnosis of AMI than conventional proteinous markers.

### Non-cardiac miRNAs

In addition to the heart- and muscle-enriched miRNAs, many other miRNAs also participate in AMI diagnosis (Table 2). miR-122 can facilitate the formation of total cholesterol and triglycerides (Esau et al., 2006). miR-126 promotes endothelial cell proliferation (Kuhnert et al., 2008). miR-199a can impair cardiomyocyte autophagy (Li Z. et al., 2017). Downregulation of miR-337 has been illustrated to protect myocadium against myocardial ischemia reperfusion injury via regulation by STAT3 (Pedretti et al., 2019). Overexpression of miR-485-5p can inhibit the mitochondrial fission and hypertrophy induced (Zhao Y.F. et al., 2017). D’Alessandra et al. (2013) revealed that miR-122, miR-126, and miR-199a were positively correlated with the risk of both UA and SA, while miR-145, miR-337-5p, and miR-485-3p were correlated only with the risk of SA or UA. miR-30c and miR-145 can prevent AMI development (Cordes et al., 2009; Duisters et al., 2009). Meder et al. (2011) found an association of elevated levels of circulating miR-30c and miR-145 with infarct size. miR-328 can prevent cell apoptosis and improve cardiac function in rats with myocardial ischemia-reperfusion injury (Ye et al., 2020). Overexpression of miR-328 in the heart can promote cardiac hypertrophy (Li C. et al., 2014). miR-134 has been revealed to play a role in the cardiogenesis and the proliferation of human cardiomyocyte progenitor cells (Wu et al., 2015). Wang R. et al. (2011) revealed a significant increase of plasma miR-328 in Chinese people with AMI compared to control subjects. He et al. (2014) also verified the potential role of circulating miR-328 as an indicator for AMI. The circulating level of miR-328 was positively related to the risk of mortality or the development of heart failure within 6 months, suggesting that miR-328 might be a prognostic biomarker for AMI. In this study, circulating miR-134 was also shown to have the same effect as miR-328 (He et al., 2014). miR-197 is associated with diabetes mellitus and platelet activation (Zampetaki et al., 2010; Willeit et al., 2013). miR-223 can promote the formation of AMI (Tabet et al., 2014). Zampetaki et al. (2012) carried out a 10-year follow-up study. They found that the increased level of miR-126 was positively correlated with AMI risk, while the decreased levels of miR-223 and miR-197 showed positive association with AMI risk. miR-21 can promote endothelial damage and dysfunction (Bonauer et al., 2009; Loyer et al., 2014). The inhibition of miR-423-5p can attenuate cardiomyocyte apoptosis and mitochondrial dysfunction (Zhu and Lu, 2019). Olivieri et al. (2013) revealed significantly increased levels of circulating miR-21 and miR-423-5p in acute NSTEMI patients. Zhang Y. et al. (2016) also reported the elevated levels of plasma miR-21 in AMI patients and demonstrated the association of miR-21 with traditional biomarkers (CK-MB and troponins). In addition, Wang et al. (2017) revealed the same increase of serum miR-21 in elderly AMI patients and the positive correlation of serum miR-21 with CK-MB and cTnI. miR-150, miR-101, miR-16 and miR-27a have been found to be associated with cardiac remodeling, myocardial fibrosis and so on (Nishi et al., 2011; Devaux et al., 2013b; Goretti et al., 2013; Chen et al., 2014). miR-486 can modulate the cardiac/skeletal muscle development (Chen et al., 2014). Devaux et al. (2013a) illustrated that circulating levels of miR-150, miR-101, miR-16, and miR-27a were associated with AMI. miR-150 and miR-101 levels were decreased while miR-16 and miR-27a levels were elevated (Devaux et al., 2013a). However, Zhang R. et al. (2015) reported an increased level of circulating miR-150 in AMI patients. They also reported the upregulated level of circulating miR-486 (Zhang R. et al., 2015). miR-34 has been elaborated to play roles in cardiac hypertrophic cardiomyopathy, pathological cardiac remodeling and atherosclerosis (Ito et al., 2010; Bernardo et al., 2012, 2014). Lv et al. (2014) identified the role of circulating miR-34a in predicting LV remodeling after AMI. In addition, they found the positive association between circulating miR-34a and the risk of mortality or heart failure, indicating its prognostic value after AMI. Yao et al. (2015) found an increased level of circulating miR-122-5p in AMI patients and a high correlation of miR-122-5p with cTnI. miRNA-210 plays a critical cardiac role in the endothelial cell response to hypoxia (Wu et al., 2009; Devlin et al., 2011). Shalaby et al. (2016) revealed the diagnostic value of serum miRNA-210 in UA and NSTEMI. MiR-221 promotes the development of AMI (Liu et al., 2012). Coskunpinar et al. (2016) reported an elevated level of circulating miR-221-3p in early AMI and found its significant correlation with troponin and LV systolic function. miR-124 has been illuminated promote cell death and apoptosis induced by myocardial ischemia-reperfusion (Liu L. et al., 2016; Liu et al., 2019). Guo et al. (2017) illustrated an upregulation of miRNA-124 and a positive correlation of miR-124 with cTnI and CK-MB in peripheral blood. Circulating miR-124 peaked earlier than cTnI and CK-MB. miR-19b has anti-atherosclerotic effect and can be anti-thrombotic protector in patients with UA (Li S. F. et al., 2014; Katta et al., 2018). miR-483-5p may be associated with coronary plaque rupture incident (Li S. F. et al., 2017; Gallo et al., 2018). Li L. et al. (2019) verified the upregulated expression of circulating miR-19b, miR-223, and miR-483-5p, which all peaked earlier than cTnI. miR-22-5p plays important roles in cardiac apoptosis, hypertrophy, fibrosis and remodeling (Huang et al., 2013; Huang and Wang, 2014). Wang et al. (2019) observed the elevated level of circulating miR-122-5p and the decreased level of circulating miR-22-5p. Further studies indicated that miR-22-5p and miR-122-5p could be combined into a panel to increase the sensitivity of the diagnosis of AMI patients (Wang et al., 2019).

Many studies on the diagnostic role of circulating miRNAs in AMI were initially based on big data analysis, but follow-up studies have also confirmed their relationship with cardiovascular disease which we will not discuss it here (Liu W. L. et al., 2015; Wei L. X. et al., 2015; Xue et al., 2015; He and Huang, 2017; Besnier et al., 2019; Che et al., 2019; Li X. D. et al., 2019; Shen et al., 2019; Zhang et al., 2019). Meder et al. (2011) identified two downregulated miRNAs, miR-1291 and miR-663b, among 121 miRNAs related to AMI through whole genome miRNA expression profiling. Among all miRNAs, miR-663b, and miR-1291 exhibited the highest sensitivity and specificity for the discrimination of AMI from healthy controls (Meder et al., 2011). However, Peng et al. (2014) found increased plasma levels of miR-663b and miR-1291with considerable diagnostic value. Jia et al. (2016) reported the considerable potential of miR-30d-5p and miR-125b-5p through microarrays in the early diagnosis of AMI. miR-30d-5p had a higher diagnostic power than cTnI. They also reported that miR-30d-5p and miR-125b-5p might have prognostic potential of AMI. Bye et al. (2016) constructed a biomarker system with miR-106a-5p, miR-424-5p, let-7g-5p, miR-144-3p, and miR-660-5p which were selected from 179 miRNAs. This system facilitated the prediction of AMI risk and improved the AMI risk stratification in healthy people (Bye et al., 2016). Wang K. J. et al. (2016) found the increased levels of plasma miR-19b-3p, miR-134-5p, and miR-186-5p in the early stage of AMI in a microarray analysis and validated the results. Plasma levels of three miRNAs were positively associated with cTnI. All their expression levels peaked earlier than cTnI.

Altogether, these miRNAs have been found to exhibit diagnostic power in AMI. However, different results for the same circulating miRNA were reported across several studies. In addition, although there were some findings, there is still a lack of prognosis analysis of these miRNAs. Moreover, the specific secretion mechanisms of most of these miRNAs are still unclear, bringing obstacles to the clinical test. Therefore, more difficulties should be overcome prior to clinical application compared with the four heart- and muscle-enriched miRNAs. Additional investigations into the diagnostic role of circulating miRNAs in AMI should be carried out to promote the clinical application.

## Clinical Challenges and Future Perspectives

Various studies have indicated the diagnostic potential of circulating miRNAs in the early diagnosis of AMI. However, given these findings, there are still many problems to be resolved before clinical application. First, the total amount of miRNAs in blood is low. Although the extraction and amplification methods have matured, situations in which certain circulating miRNA content is too low to detect occur from time to time. Therefore, more sophisticated methods should be developed. Second, no generally accepted methodology has been developed for the measurement procedures, leading to a lack of consistency across different studies. To date, the experimental procedures and methods used by different laboratories have varied. Currently, both extraction kits and TRIzol reagents are used for the circulating miRNA extraction process. Some laboratories add glycogen in Trizol method, while the other labs not. In the normalization step of circulating miRNA detection, both internal reference genes and the spiked-in genes have been used. The most widely used internal reference gene is U6 snRNA. miR-17-5p, miR-16, GAPDH, and RNU6B-2 are also used. The spiked-in reference controls generally consist of Cel-miR-39, Cel-miR-54, SV40 spike-in and HY3 molecules. Both kinds of reference genes have shortcomings. The contents of the internal reference genes are variable in different pathological circumstances, while the stability of spiked-in controls is poor in body fluids. These drawbacks might reduce the reliability of detection and increase the difficulty of comparison between similar studies. Hence, a standardized methodology that is reliable and universal remains to be developed. Third, the patient cohorts are relatively small in most studies. Only a small portion of studies are based on a cohort of more than 100 samples. As an insufficient sample size might lead to deviations in the results, larger sample sizes are necessary for future research. Fourth, the currently used qRT-PCR method is time consuming and expensive. Compared with the ELISA method for CK-MB or troponin detection, qRT-PCR costs more time and money, making rapid and cheap detection very challenging. Thus, faster and less expensive measurement methods should be developed for clinical application. Finally, the origins of circulating miRNAs are diverse (e.g., microvesicles, exosomes, Ago, and PBMCs), and our current knowledge is insufficient. Researchers have illustrated the origins of several circulating miRNAs (Condorelli et al., 2014). Nevertheless, the origins of most circulating miRNAs are unclear, making subsequent extraction and quantification difficult. Therefore, more studies should be carried out to unveil the origins of every circulating miRNA. Taken together, these challenges might be obstacles to clinical application that remain to be solved in future work.

Since the discovery of circulating miRNAs, many articles have been published on the diagnostic role of circulating miRNAs and this number will continue to grow. In recent years, scientists have also found that other non-coding nucleic acids, such as long non-coding RNA (lncRNA) and circular RNA, also exhibit diagnostic value. Their application has the same drawbacks as that of circulating miRNAs. Their circulating levels are even lower than those of circulating miRNAs, indicating a more difficult experimental operation. Therefore, the clinical application of circulating miRNAs is particularly important.

In clinical diagnosis, it is most important to obtain the diagnostic results as quickly as possible. At present, there are a variety of household rapid diagnosis instruments on the market, such as blood glucose detector and pregnancy test kit. Both diagnoses have high accuracy and are extremely fast which take only a few seconds. Both blood glucose detector and pregnancy test kit use the method of test strips. The blood glucose test needs only one drop of blood which is dropped on the test strips. The pregnancy test needs only a few drops of urine. This simple operation also offers easily recognizable diagnostic results which allows individuals to make a preliminary diagnosis at home and to monitor the state changes at any time. These two rapid diagnostic instruments are very mature and widely accepted. Perhaps we can develop an AMI clinical diagnosis kit with circulating miRNAs based on these two methods. For the formal clinical diagnosis with circulating miRNAs by hospitals, we think that diagnosis with biological probes, such as bioluminescence probes and gold nanocomposite probes, would be more rapid and accurate than RT-PCR. To achieve this, nucleic acid probes based on circulating miRNAs should be constructed first, and then test strips or detection kits based on the probes could be developed. This probe-based diagnostic approach may be a good choice for future clinical application. The diagnosis with a single circulating miRNA often has detection bias and causes diagnostic errors. A joint diagnosis system can greatly improve the efficiency and accuracy of AMI risk stratification, diagnosis and prognosis. An incorporated approach could consist of a combination of several circulating miRNAs or a combination of circulating miRNAs along with conventional proteinous indicators. Wang et al. (2013) revealed that the combination of miR-133a with cTnI significantly raised the diagnostic accuracy of coronary heart disease. Devaux et al. (2013a) found that the combination of 4 miRNAs (miR-16/27a/101/150) greatly improved the discrimination power of left ventricular (LV) contractility following STEMI. Lv et al. (2014) observed a higher value in the diagnosis and prognosis of LV remodeling after AMI when miR-208b and miR-34a were integrated. Li L. et al. (2019) reported that a panel of three miRNAs (miR-19b, miR-223, and miR-483-5p) increased the accuracy for the early diagnosis of AMI.

## Conclusion

With the development of technology and the participation of many researchers, the currently used experimental methods will be further improved and refined before clinical utility. Therefore, despite the limitations and drawbacks of circulating miRNA manipulation, we are hopeful that they will be valuable for the early diagnosis and the prognosis of AMI. Circulating miRNAs are promising biomarkers, and incorporation of circulating miRNAs into strategies for the early diagnosis and the prognosis of AMI is highly anticipated.

## Author Contributions

LZ drafted the manuscript. HD, YZ, and YW revised and edited the manuscript. WZ searched the literature. PL and LZ conceived the idea and framework of the review. All authors read and approved the final manuscript.

## Conflict of Interest

The authors declare that the research was conducted in the absence of any commercial or financial relationships that could be construed as a potential conflict of interest.

## Abbreviations

ACS: acute coronary syndrome
Ago2: argonaute protein 2
AMI: acute myocardial infarction
AP: angina pectoris
CAD: coronary artery disease
CHF: congestive heart failure
CK-MB: Creatine Kinase-MB
Ct: cycle threshold
ECGs: electrocardiograms
HDL: high-density lipoprotein
LDL: low-density lipoprotein
miRNAs: microRNAs
NPM1: nucleophosmin 1
NSTEMI: AMI without ST elevation
pre-miRNA: precursor miRNAs
pri-miRNA: primary miRNA
RISC: miRNA-induced silencing complex
SA: stable angina pectoris
SMCs: smooth muscle cells
snRNA: small nuclear RNA
STEMI: AMI with ST elevation
TnT/I: troponin T/I
UA: unstable angina.
